# 

**Published:** 1881-01

**Authors:** 


### CONTENTS.

### COMMUNICATIONS.

The Covering of Exposed Pulps........ 1
On the Comparative Value of Sulphuric Acid and Creosote in the Treatment of Alveolar Caries........ 8
The Action of Medicines........ 14
Removal of Tumors without leaving Marks........ 22
On the Behavior of Plaster of Paris in Setting........ 23

### SELECTIONS.

A Cup of Tea........ 26
Pacific Coast Fisheries........ 26
Influence of Light on Nutrition........ 27
The Way of Transgressors........ 28
Persistent Priapism........ 28
Facial Neuralgia Cured by a New Surgical Operation........ 29
Injuries to Nerves........ 30
Dangers of Anæsthesia........ 32
Supreme Court Decision........ 33

### EDITORIAL.

Specializing........ 41
Engagement Book........ 41
Ohio State Dental Society........ 42
A New Dental Journal........ 42
Mississippi Valley Dental Society........ 43
Anæsthesia—The Law........ 44
Hymenial........ 44

